# Allogeneic Hematopoietic Stem Cell Transplantation After Prior Lung Transplantation for Hereditary Pulmonary Alveolar Proteinosis: A Case Report

**DOI:** 10.3389/fimmu.2022.931153

**Published:** 2022-07-14

**Authors:** Hanne Beeckmans, Gene P. L. Ambrocio, Saskia Bos, Astrid Vermaut, Vincent Geudens, Arno Vanstapel, Bart M. Vanaudenaerde, Frans De Baets, Thomas L. A. Malfait, Marie-Paule Emonds, Dirk E. Van Raemdonck, Hélène M. Schoemans, Robin Vos

**Affiliations:** ^1^ Department of Department of Chronic Diseases and Metabolism (CHROMETA), Laboratory of Respiratory Diseases and Thoracic Surgery (BREATHE), KU Leuven, Leuven, Belgium; ^2^ Department of Internal Medicine, Division of Pulmonary Medicine, University of the Philippines – Philippine General Hospital, Manila, Philippines; ^3^ Translational and Clinical Research Institute, Newcastle University, Newcastle Upon Tyne, United Kingdom; ^4^ Department of Pediatrics, Ghent University Hospital, Ghent, Belgium; ^5^ Department of Respiratory Medicine, Ghent University Hospital, Ghent, Belgium; ^6^ Histocompatibility and Immunogenetics Laboratory, Red Cross-Flanders, Mechelen, Belgium; ^7^ Department of Thoracic Surgery, University Hospitals Leuven, Leuven, Belgium; ^8^ Department of Hematology, Bone Marrow Transplant Unit, University Hospitals Leuven, Leuven, Belgium; ^9^ Department of Public Health and Primary Care, Academic Centre for Nursing and Midwifery, Katholieke Universiteit (KU) Leuven, Leuven, Belgium; ^10^ Department of Respiratory Diseases, University Hospitals Leuven, Leuven, Belgium

**Keywords:** pulmonary alveolar proteinosis, lung transplantation, bone marrow transplantation, allogeneic hematopoietic stem cell transplantation, rejection

## Abstract

Pulmonary alveolar proteinosis (PAP) is a rare, diffuse lung disorder characterized by surfactant accumulation in the small airways due to defective clearance by alveolar macrophages, resulting in impaired gas exchange. Whole lung lavage is the current standard of care treatment for PAP. Lung transplantation is an accepted treatment option when whole lung lavage or other experimental treatment options are ineffective, or in case of extensive pulmonary fibrosis secondary to PAP. A disadvantage of lung transplantation is recurrence of PAP in the transplanted lungs, especially in hereditary PAP. The hereditary form of PAP is an ultra-rare condition caused by genetic mutations in genes encoding for the granulocyte macrophage-colony stimulating factor (GM-CSF) receptor, and intrinsically affects bone marrow derived-monocytes, which differentiate into macrophages in the lung. Consequently, these macrophages typically display disrupted GM-CSF receptor-signaling, causing defective surfactant clearance. Bone marrow/hematopoietic stem cell transplantation may potentially reverse the lung disease in hereditary PAP. In patients with hereditary PAP undergoing lung transplantation, post-lung transplant recurrence of PAP may theoretically be averted by subsequent hematopoietic stem cell transplantation, which results in a graft-versus-*disease (PAP)* effect, and thus could improve long-term outcome. We describe the successful long-term post-transplant outcome of a unique case of end-stage respiratory failure due to hereditary PAP-induced pulmonary fibrosis, successfully treated by bilateral lung transplantation and subsequent allogeneic hematopoietic stem cell transplantation. Our report supports treatment with serial lung and hematopoietic stem cell transplantation to improve quality of life and prolong survival, without PAP recurrence, in selected patients with end-stage hereditary PAP.

## Introduction

Pulmonary alveolar proteinosis (PAP) is a rare, diffuse lung disorder characterized by accumulation of surfactant in the small airways (1–4). PAP has a prevalence of 6-7 cases per million population worldwide, and >90% of PAP cases are of autoimmune etiology, in which a high level of autoantibodies against granulocyte–macrophage colony-stimulating factor (GM-CSF) neutralize the biologic activity of GM-CSF, thereby causing poor surfactant clearance. Mutations in the heterodimeric GM-CSF receptor α or β chains (CSF2RA, CSF2RB), the surfactant proteins B or C, ATP-binding cassette subfamily A member 3 (ABCA3), and thyroid transcription factor-1 were identified causing hereditary PAP, which can be considered the archetype of ultra-rare diseases (2, 5, 6). Without GM-CSF or in case of disrupted GM-CSF receptor-signaling, alveolar macrophages display reduced surfactant clearance, which accumulates and impairs alveolar gas exchange. Secondary PAP on the other hand, is caused by an underlying disease affecting alveolar macrophage function, which includes surfactant catabolism disorders. These encompass hematological conditions, malignancies, infectious diseases, immune deficiency syndromes, and toxic inhalation exposures (2).

PAP is diagnosed based on a compatible history, typical radiologic features, bronchoalveolar lavage cytology and/or lung biopsy results, and compatible biomarkers. Clinical presentation of PAP is nonspecific, and may vary in severity and clinical presentation. Onset may present insidiously, obscuring timely diagnosis, often only taking place months to years after disease onset. Common symptoms include exertional dyspnea, cough, fatigue, and weight loss (2, 4). Serum GM-CSF antibody testing is a first, noninvasive diagnostic test and can diagnose ~90% of cases of PAP. This approach avoids the need for an invasive procedure, such as lung biopsy, in the setting of a compatible history and typical imaging findings. Chest radiograph typically shows bilateral symmetrical opacities in the mid and lower lung fields. Classic features on high resolution computed tomography are homogenously distributed ground-glass opacities, and presence of thickened intralobular structures and interlobular septa in typical polygonal shapes, superimposed on ground glass opacities, referred to as “crazy-paving” (7, 8). Bronchoalveolar lavage fluid shows a milky, turbid, appearance and thick sediment; which is abundant PAS-positive proteinaceous material in and around alveolar macrophages (2, 9, 10). Pulmonary function tests usually demonstrate a reduction in the diffusing capacity for carbon monoxide that may be isolated or accompanied by a restrictive ventilatory defect (11). In contrast to autoimmune PAP, in which anti-GM-CSF antibodies are present, these are absent in hereditary PAP, in which abnormal GM-CSF receptor function is documented using genetic sequencing to confirm the presence of recessive genetic variants (2, 11). Supplementary laboratory testing is dependent on the exclusion of causes for secondary PAP. The definitive diagnosis of PAP is based on the presence of histopathological features of amorphous, Periodic Acid-Schiff (PAS)-positive lipoproteinaceous material (surfactant) in the terminal bronchioles and alveoli on transbronchial or surgical biopsy (7).

If untreated or in case of ineffective treatment, primary PAP will result in respiratory insufficiency and death, due to impaired alveolar gas exchange and/or fibrotic evolution of PAP. Extensive interstitial fibrotic disease, however, only occurs rarely in PAP (12). In these patients, lung transplantation may be an acceptable treatment option to improve quality of life and prolong survival. A disadvantage for lung transplantation, however, is recurrence of PAP in the transplanted lungs, particularly in hereditary PAP, as *donor* alveolar macrophages will be gradually replaced by *recipient* (GM-CSF receptor-deficient) monocyte-derived alveolar macrophages during the years following lung transplantation, although long-term persistence of donor alveolar macrophages has recently been described (13–16). Mice models have demonstrated that the hereditary form of PAP affects bone-marrow derived monocytes, and that bone marrow/hematopoietic stem cell transplant may potentially reverse the disease (17). Hematopoietic stem cell transplantation following prior lung transplantation may theoretically result in a graft-versus-*disease (PAP)* effect that would prevent post-lung transplant recurrence of PAP, and thus could improve long-term outcome of patients with hereditary PAP, in case no other effective treatment options are available.

We herein present a case of hereditary PAP who underwent bilateral lung transplantation for end-stage respiratory failure due to PAP-induced pulmonary fibrosis, followed by subsequent allogeneic hematopoietic stem cell transplantation (allo-HSCT), and report on the successful long-term post-transplant outcome of this unique case.

## Case Description

A male patient was diagnosed with PAP in 2000, at the age of 35 months. At that moment interstitial lung disease was diagnosed following respiratory complaints of coughing, dyspnea and fever, with persistent opacities on chest X-ray despite antibiotic treatment with clarithromycin and amoxicillin-clavulanate. Prior to this, the parents had noted chronic tachypnea since more than 6 months. Clinically, insufficient weight gain (percentile 3-25), and pectus excavatum were noticed. Chest computed tomography demonstrated bilateral ground glass opacities, with sparing of the apical and peripheral lung fields. Histologic assessment of an open lung biopsy of the left lung lower lobe demonstrated the presence of alveolar lipoproteinosis (**Supplementary Figure 1**). Additional scanning electron microscopy confirmed the presence of lamellar surfactant bodies in the alveoli and in the cytoplasm of alveolar type II cells, consistent with the diagnosis of PAP.

Additional investigations in 2008 demonstrated no evidence for surfactant protein B deficiency (i.e. no mutation of SFTPB indel g.1549C > GAA (121ins2)), nor the presence of anti-GM-CSF antibodies. In 2010, at the age of 13, genetic analysis revealed a large homozygous deletion of chromosomes Xp22.3 and Yp11.3, affecting the complete CSF2RA gene (13 exons), the complete CRLF2 gene (6 exons) situated on its telomeric side, as well as exons 1-3 and most exons 1-5 of the IL3RA gene situated on its centromeric side, resulting in a complete deficiency of GM-CSF receptor α subunit. (18, patient G is the current patient) Both parents were heterozygous carriers of the genetic defects in the pseudoautosomal region 1 (PAR1) on the distal end of the X and Y chromosomes, respectively, confirming the diagnosis of hereditary PAP. At that time, his 4-year younger sister was not tested for the mutation, but currently has no evidence for PAP, at the age of 19.

**Figure 1:**
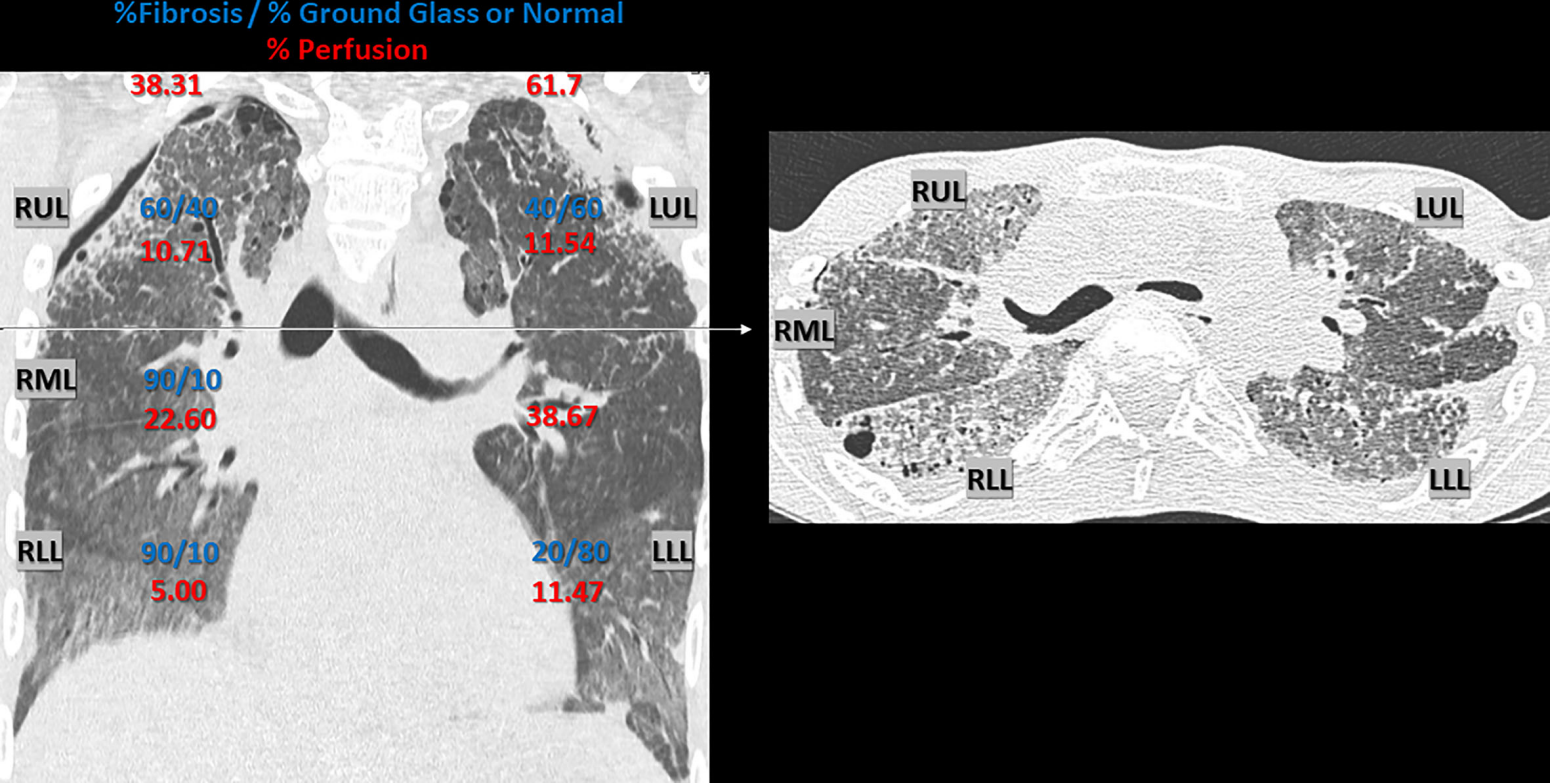
Chest CT Scan 1 year pre-lung transplant. Chest CT Scan 1 year pre-lung transplant (2016), with representative coronal and axial section, demonstrating interstitial lung disease, with parenchymal fibrosis and ground glass opacities, due to alveolar lipoproteinosis caused by hereditary pulmonary alveolar proteinosis. Radiologic signs of irreversible fibrosis included thickening of intra- and interlobar septae (reticulation), traction bronchiectasis and honeycombing. Architectural distortion was more prominent in the anterior en apical lung fields compared to the postero-basal lung fields. A secondary spontaneous partial pneumothorax was present, situated bilateral apico-lateral (dehiscence: left side 8 mm and right side 5 mm). An estimate of the ratio (%) fibrosis *vs*. ground glass opacification or normal appearing lung parenchyma per lobe is given in blue (visual scoring by a skilled chest radiologist with expertise in pulmonary fibrosis). The respective perfusion (%) of each lung and individual lobe is given in red, illustrating regional differences due to hypoxic vasoconstriction. Perfusion was measured using technetium-99m radioisotope (122.0 MBq, 3.30 mCI) and Pulmonics software. RUL, right upper lobe; RML, right middle lobe; RLL, right lower lobe; LUL, left upper lobe; LLL, left lower lobe.

**Figure 2:**
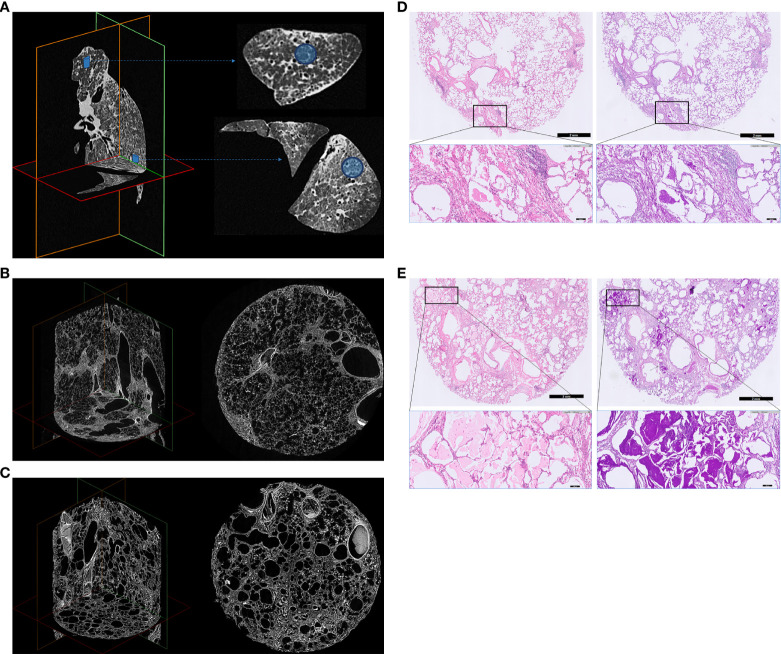
Explanted left lung *ex vivo* CT, micro-CT and histology. **(A)** *Ex vivo* CT. **(B)** Upper lobe lung tissue core micro-CT **(C)** Lower lobe lung tissue core micro-CT. **(D)** Upper lobe lung tissue core histology. **(E)** Lower lobe lung tissue core histology. Explanted left lung *ex vivo* CT **(A)**, micro-CT images **(B, C)** and histology **(D, E)** of 2 lung tissue cores demonstrating severe interstitial lung disease, with heterogenous distribution, resulting in architectural deformation of the lung ultrastructure, as a result of end-stage hereditary pulmonary alveolar proteinosis. The lung was recovered at lung transplantation, and processed for imaging and histology as previously described (18, 19). Multidetector CT was obtained using a Siemens Somatom (120 kV, Br59d reconstruction kernel, pixel spacing: 0.7 mm, 1 mm slice thickness) to determine the localization of cores in the lung. Afterwards, the lung was sawed in sections of 2 cm using a bandsaw, and cores of 1.4 cm diameter were taken in the apex and basal part using a hole saw. These lung tissue cores were chemically dried using hexamethyldisilazane (Sigma-Aldrich, USA) and scanned with a Skyscan 1272 microCT scanner (50 kV, 200 µA, voxel size: 8.1 µm, Bruker, Kontich, Belgium). Animated videos of the *in vivo* CT prior to lung transplantation and the *ex vivo* CT and micro-CT of the explanted left lung are presented in **Supplemental Figure 2**. For histology, tissue cores were routinely formalin-fixed and paraffin embedded. Sections of 5 µm were subsequently stained with Hematoxylin and Eosin (H&E) using an automated H&E platform (CoverStainer, Dako Agilent) and with Periodic Acid Schiff (PAS) using an automated special staining system (Artisan Link Pro Special Staining System, Dako Agilent). Corresponding histological images are shown from upper/lower lobe tissue core: hematoxylin eosin (H&E) staining (left), and Periodic Acid Schiff (PAS) staining after diastase digestion (right) (200x total magnification). The lung parenchyma shows an overt fibrotic remodeled alveolar parenchyma, with thickening of intra- and interlobar septae (reticulation), traction bronchiectasis/bronchiolectasis, honeycombing and prominent filling of the alveolar spaces with a granular eosinophilic proteinaceous material, which stains strongly PAS positive. Architectural distortion due to these histologic changes was observed both in the apical and basal lung fields/cores.

The patient underwent at least annual sequential whole lung lavages, which started in 2000 when he was 3 years old, and were continued throughout childhood until 2015, at the age of 17, during which period a total number of 32 procedures were performed. During this 14-year treatment period, a few mild respiratory infections occurred during early childhood (age <8), necessitating antibiotic treatment, however the patient remained free from severe respiratory infections necessitating hospitalization or respiratory tract colonization with gram negative bacteria or fungi, as lung lavage cultures always remained negative. Since 2010, at the age of 13, his pulmonary status gradually worsened, with progressive restrictive lung disease and radiologic evidence of pulmonary fibrosis, cachexia, and hypoxic and hypercapnic respiratory failure, finally necessitating oxygen treatment as of 2016, and nightly non-invasive ventilation as of 2017. Eventual findings were compatible with progressive interstitial lung disease with extensive lung fibrosis (**Figures 1** and **2**, and **Supplementary Figure 2**). During the course of his disease and the treatment with whole lung lavages, several international experts were consulted regarding possible other treatment options, such as inhaled GM-CSF (2009), bone marrow transplantation (2012), gene therapy (2012), pulmonary macrophage transplantation therapy (2017), or vector-induced genetic correction of autologous macrophages (2017), none of which was deemed accessible, feasible or safe at that time. Finally, he was referred for lung transplantation in 2016, at the age of 18. His respiratory condition at referral for lung transplant is summarized in **Supplementary Table 1**. At that time, azithromycin (250mg/day, three times a week) was started for prevention of infections, and for its anti-inflammatory properties on lung epithelial cells, as well as to lower pro-inflammatory gene expression and to improve phagocytosis by alveolar macrophages (20). Later, ambroxol as mucolytic drug was associated (30 mg, three times a day) (21). Pulmonary rehabilitation and hypercaloric, protein-rich diet were initiated, in order to optimize the patient’s physical condition prior to transplant.

Because of the severity of his underlying lung disease and the extent of fibrosis, hematopoietic stem cell transplantation alone was considered to be both ineffective, as this would not resolve the pulmonary fibrosis, and unsafe, given the risk for pulmonary complications associated with the conditioning-regimen prior to stem cell transplantation. On the other hand, lung transplantation alone was considered to be a suboptimal option to improve the patient’s long-term outcome because of the high risk for post-lung transplant relapse of the disease, therefore subsequent stem cell transplantation was deemed necessary. Since this could not be achieved by using stem cells obtained from the same (lung) donor for logistical reasons, planning for allo-HSCT was initiated.

As stem cell donors, neither his parents, nor sister were fully Human Leukocyte Antigen (HLA)-compatible (**Table 1**), therefore a Matched Unrelated Donor (MUD) search was performed, aimed at finding a HLA-identical donor, which would limit the risk for later graft-versus-host disease (GvHD), and likely also the risk for lung graft rejection following (non-HLA compatible) lung transplantation. MUD search identified two fully HLA-compatible MUD. As donor macrophages remain in the lung allograft for up to one year, after which they are progressively replaced by macrophages of receptor-origin, the time frame for allo-HSCT was set at 9 to 11 months post-lung transplantation, at which point the post-transplant infectious risk and immunosuppressive therapy was estimated to safely allow the conditioning-regimen and stem cell transplant procedure, with expected recovery of the bone marrow function by 1 year post-lung transplant.

**Table 1:** HLA profile of index case, relatives and donors.

HLA	Haplotype	A	B	C	DRB1	DQB1	DPB1	HLA match	ABO	CMV IgG	Age	Donor
**Patient**	A	32:01	40:02	02:02	11:01	03:01			O pos	Pos	18	
** **	C	30:02	18:01	05:01	03:01	02:01						
**Mother**	A	32	61	02	11	07		5/10				
** **	B	68	60	03	13	06						
**Father**	C	30	18	05	03	02		5/10				
** **	D	68	55	03	04	08						
**Sister**	A	32	61	02	11	07		5/10				
** **	D	68	55	03	04	08						
**MUD1**		30:02/32:01	18:01/40:02	02:02/05:01	03:01/11:01	02:01/03:01		10/10	O pos	Neg	21	MUD
**MUD2**		30:02/32:01	18:01/40:02	02:02/05:01	03:01/11:01	02:01/03:01		10/10	O pos	Neg	31	Back-up
**Lung Donor**		68:02/25:01	14:02/18:02	07:01/08:02	11:04/13:03	03:01/NA	02:01/04:01	3/10	O pos	Neg	36	DBD

HLA profile of index case, relatives and donors. Matching haplotypes are indicated in color (A: red, C: blue).

HLA, Human leucocyte antigen; ABO, ABO blood group type; CMV, Cytomegalovirus; IgG, immunoglobulin G; MUD, matched unrelated donor; DBD, donor after brain death.

Pre-transplant evaluation revealed no major contraindications for lung transplantation or allo-HSCT and after a waiting list time of 342 days, the patient underwent successful bilateral lung transplantation in 2017, at the age of 19. The lung donor was a 36-year old male donor after brain death (blood type O positive, Cytomegalovirus negative), and the procedure was performed under veno-venous (left femoral outflow, right femoral inflow) extracorporeal membrane oxygenation support, which was initiated prior to bilateral anterior thoracotomy because of hypercapnia. Bilateral pneumonectomy was laborious, due to important pleural adhesions and bleeding. Implantation times were 77 minutes (right) and 81 minutes (left), and cold ischemic times were 463 minutes (right) and 728 minutes (left), respectively. Total duration of extracorporeal support was 507 minutes, and total duration of the surgery was 643 minutes.

Methylprednisolone 500 mg was given intraoperatively, post-transplant induction immunosuppression consisted of rabbit antithymocyte globulin (3 mg/kg/day, for 3 days), and maintenance immunosuppression of methylprednisolone, tacrolimus and mycophenolate mofetil, as per routine protocol. Standard infectious prophylaxis with nebulized amphotericin B lipid complex (1 month), ganciclovir (6 months), azithromycin (life-long), and trimethoprim/sulfamethoxazole (life-long) was utilized. The early post-transplant period was characterized by a right-sided hemothorax, for which a surgical revision was performed on post-operative day 4, and a respiratory infection with methicillin-sensitive *Staphylococcus aureus*, treated with flucloxacillin. Because of prolonged delirium and hallucinations, tacrolimus was switched to cyclosporin A on day 15, with good clinical evolution thereafter. He was discharged home on day 30. Apart from one episode of probable invasive pulmonary aspergillosis (*Aspergillus fumigatus*) at 6 months post-transplant, which was treated with voriconazole (12 weeks), there was an uneventful clinical course until the patient underwent subsequent stem cell transplantation.

**Figure 3:**
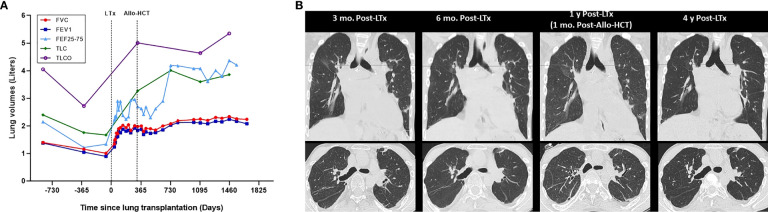
Evolution of pulmonary function and chest CT over time. **(A)** Pulmonary function. **(B)** Chest CT. Evolution of pulmonary function over time in relation to lung transplantation (LTx) and allogeneic hematopoietic stem cell (Allo-HCT) for hereditary pulmonary alveolar proteinosis **(A)**, which are indicated by dotted lines. Dots represent individual measurements of lung volumes (Liters), with connecting lines between measurements showing the trend of individual lung function parameters over time. Evolution of chest computed tomography (CT) function over time **(B)**, with representative inspiratory phase images at 3 months, 6 months 1 year and 4 years post-lung transplantation (LTx). Allogeneic hematopoietic stem cell (Allo-HCT) was performed 11 months after prior lung transplantation. At 4.5 years after lung transplantation there is currently no evidence for chronic lung allograft dysfunction, graft-versus-host disease, or recurrence of hereditary pulmonary alveolar proteinosis. FVC, forced vital capacity; FEV1, forced expiratory volume in one second; FEF25-75, forced expiratory flow at 25–75% of forced vital capacity; TLC, total lung capacity; TLCO, transfer capacity of the lung, for the uptake of carbon monoxide (CO) (also called diffusing capacity for carbon monoxide).

Allo-HSCT was performed 336 days (11 months) after prior lung transplantation. The stem cell donor was a 21-year old, male, 10/10 HLA-identical donor (blood type O positive, Cytomegalovirus negative). Reduced-intensity conditioning regimen for allo-HSCT consisted of fludarabine (180 mg/m², day -6 until day -1); busulfan (8mg/kg, day -4 and day -3) and rabbit antithymocyte globulin (5 mg/kg, day -2 and day -1) (revised Slavin scheme) (22). A total number of 6.22 x10^6^ peripheral blood CD34+ cells/kg were administered. Neutrophil engraftment occurred on day 19, and donor chimerism of >95% was documented on day 30. Acute GvHD prophylaxis consisted of mycophenolate mofetil and cyclosporin A, which were continued immediately after the stem cell transplant procedure and were not stopped prior to the transplant-procedure. Infectious prophylaxis consisted of levofloxacin and acyclovir until engraftment, and posaconazole (12 weeks). The early post-transplant period was characterized by neutropenic fever, treated with meropenem, besides nausea, anorexia and pruritus, treated symptomatically. No bacteremia or pulmonary complications occurred. He was discharged home on day 20. At 1 month after stem cell transplantation, cytomegalovirus reactivation occurred, for which acyclovir was switched to valganciclovir (4 months), which resulted in by asymptomatic neutropenia, treated by intermittent use of G-CSF. Intravenous immunoglobulin substitution (life-long) was initiated for secondary hypogammaglobulinemia. There was an uneventful clinical course during the following years, except for intermittent Epstein-Barr-virus (EBV) reactivation without evolution to post-transplantation lymphoproliferative disease, but otherwise no respiratory tract infections, acute pulmonary rejection episodes, development of chronic lung allograft dysfunction, pulmonary or extra-pulmonary GvHD, or PAP-recurrence at present, 4.5 years after lung transplantation. (**Figure 3**) Meanwhile, the patient has an excellent quality of life, is actively performing sports (fitness, paddle, cycling), has successfully finished his University Master’s studies, obtained a Postgraduate Degree, and is at present full-time working as Data Analyst. He is currently maintained on methylprednisolone, cyclosporine A, and mycophenolate mofetil; and azithromycin and trimethoprim/sulfamethoxazole for prophylaxis.

## Discussion

The current report describes the successful long-term post-transplant outcome of a unique case of end-stage respiratory failure due to hereditary PAP-induced pulmonary fibrosis, successfully treated by bilateral lung transplantation and subsequent fully HLA matched unrelated allo-HSCT. Our report thus supports treatment with serial lung and stem cell transplantation to improve quality of life and prolong survival, without PAP recurrence, in selected patients with end-stage hereditary PAP.

Various forms and stages of PAP may require different therapeutic options. Nevertheless, symptomatic treatment with whole lung lavage is the current standard of care for PAP, as it can be performed in the various forms of the disease, irrespective of the pathophysiological mechanisms involved (2, 23, 24). Attenuating the natural evolution of the disease by removal of alveolar proteinosis with lung lavage may significantly improve alveolar-arterial oxygen gradient, vital capacity, total lung capacity, and diffusing capacity, clinically resulting in fewer symptoms and better quality of life: reduced dyspnea, less need for supplementary oxygen, and improved exercise capacity (i.e. distance walked). However, these effects are often only transient, since the recurrence rate of PAP after whole lung lavage ranges from 28-80% (23). Aside from disease recurrence, issues of concern moreover include lack of standardization of the procedure, and a plateau of the therapeutic benefit over time due to alveolar scarring and distortion (interstitial fibrosis), which occurs secondary to alveolar collapse. Other therapeutic options, such as supplementation of exogenous GM-CSF or strategies aimed at reducing the levels of the autoantibodies (i.e. B-cell directed therapy with rituximab in autoimmune PAP, plasmapheresis, or combining these therapies with whole lung lavage) have been reported for the management of difficult autoimmune PAP cases (2, 3).

In patients with hereditary PAP, caused by recessive mutations in GM-CSF receptor subunit α/β genes, defective GM-CSF receptor-signaling leads to impaired surfactant clearance by alveolar macrophages resulting in alveolar surfactant accumulation. In these patients, alveolar macrophages are unable to bind GM-CSF and the ensuing defective GM-CSF signaling subsequently results in functional defects in phagocytosis (5). Therefore, GM-CSF administration is ineffective, and other therapeutic options are necessary to cure this rare, life-threatening lung disease. Theoretically, bone marrow/allogeneic stem cell transplantation, gene therapy, pulmonary macrophage transplantation therapy, or vector-induced genetic correction of autologous macrophages may be options to correct the underlying mutations driving disease onset and progression in hereditary PAP. However, none of these options have currently been standardized as treatment in humans.

Lung transplantation has been performed in cases in which other treatments were ineffective and/or with disease progression. This is an option for severe, treatment-refractory cases; especially if there is irreversible, extensive pulmonary fibrosis, as in our case (2, 13, 14, 23). A disadvantage of lung transplantation, however, is recurrent PAP in the transplanted lungs, which may occur withing the first years post-transplant (13, 14). Especially in hereditary PAP, due to germ line mutations in the GM-CSF receptor gene, it is expected that the disease might recur early after lung transplantation, since the mutation also affects bone marrow-derived monocytes which ultimately contribute to the alveolar macrophage pool. Indeed, in a patient with a nonsense mutation in CSF2RB, disease recurrence was demonstrated 9 months post-lung transplantation, at which moment donor-origin alveolar macrophages had been almost completely replaced with recipient-origin macrophages (13). As far as we know, this was the only reported case of lung transplantation in documented hereditary PAP, again highlighting the unique situation of our case.

Under homeostatic conditions, alveolar macrophages of the lung are long-lived and self-renew to maintain their population without contribution from circulating adult bone marrow-derived monocytes. However, in response to inflammatory stimuli and lung injury, it has been demonstrated that recruited bone marrow derived-monocytes can differentiate into macrophages in the lung, and acquire the tissue-resident alveolar macrophage phenotype. These alveolar macrophages are long-lived, may self-renew and are critical for tissue homeostasis, host defense, clearance of surfactant and cell debris, pathogen recognition, initiation and resolution of lung inflammation, and repair of damaged tissue (25). This pathway highlights two important issues to take into account regarding hereditary PAP.

First, the possibility that allo-HSCT may reverse the lung disease, by a graft-versus-*disease (PAP)* effect, as was demonstrated in a murine model (17). Of note, since it concerns a non-malignant disease, reduced-intensity conditioning for (nonmyeloablative) bone marrow transplantation, which is generally less toxic and better tolerated, may suffice to allow for successful stem cell engraftment, as demonstrated in our case. However, allo-HSCT still carries a high risk for complications, such as infection, graft-versus-host disease (GVHD) and drug toxicity. This is illustrated by a case of a 18-year old boy with the CSF2RA mutation who died 8 months post-transplant of pneumoniae and sepsis while under intense immunosuppression due to GVHD (26). Another 4-year old girl with hereditary PAP due to genetic deficiency of GM-CSFRα, who underwent allo-HSCT with an HLA-matched unrelated donor, died from a respiratory infection 4 weeks after transplant, before recovery of immune competency (27). Similarly, a 6-year old girl with hereditary PAP due to genetic deficiency of GM-CSFRα, who underwent allo-HSCT from a 10/10 HLA-matched unrelated donor, suffered from life-threatening acute GVHD (gut, skin), rapamycin-induced pulmonary toxicity, and chronic GVHD (eyes, lungs) at 40 months post-transplant (28). These are the only reported cases of allo-HSCT in proven hereditary PAP, as far as we know. Of note, recurrence of autoimmune PAP and development of secondary PAP have also been described as a late complication of both allo-HSCT (29) or lung transplantation (30), emphasizing the need for post-transplant follow-up.

Secondly, the fact that following lung transplantation *donor* alveolar macrophages in the lung graft are gradually replaced and repopulated by *recipient* alveolar macrophages over time, *via* bone marrow-derived monocytes (15). This also illustrates the need to consider a congenital defect in GM-CSF signaling as a cause of PAP before performing a lung transplantation, thus the need for genetic evaluation, and the possibility of additional allo-HSCT as a long-term curative treatment for PAP in these patients (2, 17, 23, 31). The timing of allo-HSCT, however, is an important issue, given the critical period of progressive replacement of donor-derived alveolar macrophages by recipient-derived macrophages during the first year post-lung transplant, and thus the associated risk of PAP recurrence. Preservation of post-transplant lung allograft function/quality by preventing disease recurrence is therefore perhaps preferable, rather than waiting for disease recurrence, in order to avoid deterioration of graft function, possible graft failure and the need for lung retransplantation, given the scarcity of donor lungs. Postponing allo-HCT until PAP reappears may furthermore be less preferable given the associated risk of respiratory infections (due to alveolar atelectasis/macrophage dysfunction, or procedure-related infections in case whole lung lavages are needed), in these immunocompromised patients.

Our report hence highlights a successful case of lung transplantation followed by allo-HSCT in a patient with end-stage pulmonary fibrosis due to hereditary PAP, which strategy may perhaps also apply to selected patients with other genetic disorders affecting both lungs and bone marrow, such as other genetic causes of PAP (6, 32, 33), dyskeratosis congenita (34) (which is characterized by lung fibrosis and bone marrow failure), or rare primary immunodeficiency diseases (which may cause infection-related lung remodeling leading to respiratory insufficiency) (35). The good long-term outcome of our unique case supports this approach with the aim of improving quality of life and survival, while preventing disease recurrence, in selected patients with hereditary PAP, while awaiting novel treatment options to become available in the future.

## Data Availability Statement

The raw data supporting the conclusions of this article will be made available by the authors, without undue reservation.

## Ethics Statement

The local Ethics Committee of UZ/KU Leuven approved the current study (S59591) and per institutional protocol the patient provided written informed consent at time of listing for lung transplantation and prior to allogeneic stem cell transplantation to access and use his clinical and biobanked data for scientific research purposes (S51577 and TR-00ALG1-001). Informed consent was obtained from the patient for the current case report.

## Author Contributions

HB: data collection, data curation, final draft preparation, and review and editing; GA: data collection, data curation, preliminary draft preparation, and review and editing; SB: final draft preparation and review and editing; AV: data collection, data curation, visualization, final draft preparation, and review; VG: data collection, data curation, visualization, final draft preparation, and review and editing; AV: data collection, data curation, visualization, final draft preparation, and review and editing; BV: data collection, data curation, visualization, final draft preparation, and review and editing; FB: final draft preparation and review and editing; TM: final draft preparation and review and editing; M-PE: data collection, data curation, visualization, final draft preparation, and review and editing; DR: data collection, data curation, final draft preparation, and review and editing; HS: data collection, data curation, final draft preparation, and review and editing; RV: conceptualization, methodology, data collection, data curation, visualization, final draft preparation, review and editing, and funding. All authors contributed to the article and approved the submitted version.

## Funding

GA is a recipient of the European Respirology Society (ERS) Clinical Training Fellowship (2018) and is included under the Scientifically Developing Countries Program. HB is supported by a pre-doctoral grant of KU Leuven (DB/21/012/bm). SB is supported by the Paul Corris International Clinical Research Training Scholarship. VG is s supported by a PhD research fellowship of the Research Foundation-Flanders (FWO) (11L9822N). BV is supported by a research grant form KU Leuven (C24/050). DR is supported by the Broere Charitable Foundation. HS is supported by a Start-Up fund Grant from UZ Leuven Klinische onderzoeks- en opleidingsraad (KOOR). RV is supported by a research fellowship as Senior Clinical Research Fellow of Research Foundation-Flanders (FWO) and by research project of the Research Foundation-Flanders (FWO) (G060322N).

## Conflict of Interest

The authors declare that the research was conducted in the absence of any commercial or financial relationships that could be construed as a potential conflict of interest.

## Publisher’s Note

All claims expressed in this article are solely those of the authors and do not necessarily represent those of their affiliated organizations, or those of the publisher, the editors and the reviewers. Any product that may be evaluated in this article, or claim that may be made by its manufacturer, is not guaranteed or endorsed by the publisher.

## Supplementary Material

Supplementary Figure 1 Histology of open lung biopsy of the left lung lower lobe demonstrating the presence of alveolar lipoproteinosis. **(A)** Representative images from diagnostic lung wedge biopsy (upper: stained with hematoxylin eosin (HE); lower: stained with Periodic Acid Schiff (PAS) after diastase digestion, 200x total magnification). Presence of prominent airspace filling by a granular eosinophilic proteinaceous material with a background of inconspicuous normally preserved alveolar septa without interstitial inflammation. This eosinophilic proteinaceous material stains moderately positive with PAS after diastase digestion. **(B)** Representative images from diagnostic lung wedge biopsy: hematoxylin eosin (H&E) staining **(A)**, and Periodic Acid Schiff (PAS) staining after diastase digestion **(B)** (200x total magnification). Presence of prominent airspace filling by a granular eosinophilic proteinaceous material with a background of inconspicuous normally preserved alveolar septa without interstitial inflammation. This eosinophilic proteinaceous material stains positive with PAS after diastase digestion.

Supplementary Figure 2 Explanted left lung CT and micro-CT. **(A)** (*In vivo* chest CT pretransplant 2016.avi). **(B)** (Explanted Left Lung CT.mp4). **(C)** (Upper Lobe Tissue Core microCT.mp4). **(D)** (Lower Lobe Tissue Core microCT.mp4). Animated videos of the *in vivo* chest CT in 2016 (one year before lung transplantation) **(A)**, *ex vivo* CT of the explanted left lung **(B)** and *ex vivo* micro-CT of the 2 lung tissue cores obtained from the upper **(C)** and lower lobe **(D)** demonstrating severe interstitial lung disease, with heterogenous distribution, resulting in architectural distortion of the lung ultrastructure, as a result of end-stage hereditary pulmonary alveolar proteinosis. Radiologic signs of irreversible fibrosis included thickening of intra- and interlobar septae (reticulation), traction bronchiectasis and honeycombing.

Supplementary Table 1 Respiratory status at referral for lung transplantation. Pulmonary function (spirometry, body plethysmography, diffusing capacity), exercise capacity test (6 minute walk distance (6MWD), cycloergometry) and arterial blood gas evaluation at referral for lung transplantation in 2016, demonstrating severe restrictive pulmonary dysfunction and decreased oxygen uptake, impaired exercise capacity and hypoxic, hypercapnic respiratory insufficiency.

## Abbreviations:

Pred., predicted value; Meas., measured value; %Pred, percentage of predicted value; FVC, forced vital capacity; FEV1, forced expiratory volume in one second; PEF, peak expiratory flow; FEF25%, forced expiratory flow at 25% of forced vital capacity; FEF50%, forced expiratory flow at 50% of forced vital capacity; FEF75%, forced expiratory flow at 75% of forced vital capacity, FEF25-75%, forced expiratory flow at 25–75% of forced vital capacity; VC, vital capacity; RV, residual volume; TGV, thoracic gas volume; TLC, total lung capacity; TLCO, transfer capacity of the lung, for the uptake of carbon monoxide (CO) (also called diffusing capacity for carbon monoxide); KCO, carbon monoxide transfer coefficient; 6MWD, 6 minute walking distance; Satc O2, transcutaneous oxygen saturation; VO2max, maximal oxygen consumption.
