# Supplementary figures and images for: Allogeneic Hematopoietic Stem Cell Transplantation After Prior Lung Transplantation for Hereditary Pulmonary Alveolar Proteinosis: A Case Report
Source: Front Immunol. 2022 Jul 14;13:931153. doi: 10.3389/fimmu.2022.931153 (PMC9344132; doi:10.3389/fimmu.2022.931153)

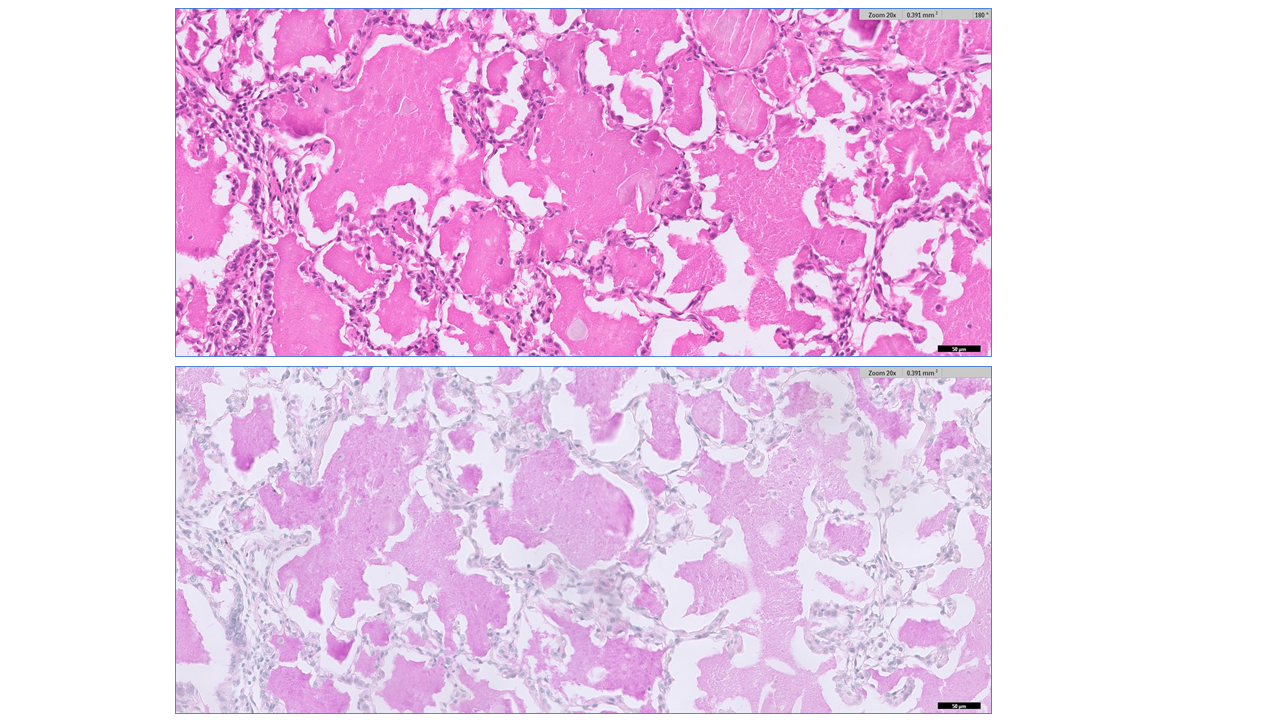

Supplement: Supplementary Figure 1 — Histology of open lung biopsy of the left lung lower lobe demonstrating the presence of alveolar lipoproteinosis. (A) Representative images from diagnostic lung wedge biopsy (upper: stained with hematoxylin eosin (HE); lower: stained with Periodic Acid Schiff (PAS) after diastase digestion, 200x total magnification). Presence of prominent airspace filling by a granular eosinophilic proteinaceous material with a background of inconspicuous normally preserved alveolar septa without interstitial inflammation. This eosinophilic proteinaceous material stains moderately positive with PAS after diastase digestion. (B) Representative images from diagnostic lung wedge biopsy: hematoxylin eosin (H&E) staining (A), and Periodic Acid Schiff (PAS) staining after diastase digestion (B) (200x total magnification). Presence of prominent airspace filling by a granular eosinophilic proteinaceous material with a background of inconspicuous normally preserved alveolar septa without interstitial inflammation. This eosinophilic proteinaceous material stains positive with PAS after diastase digestion. [file Image_1.tif]
