# Supplementary material for: Allogeneic Hematopoietic Stem Cell Transplantation After Prior Lung Transplantation for Hereditary Pulmonary Alveolar Proteinosis: A Case Report
Source: Front Immunol. 2022 Jul 14;13:931153. doi: 10.3389/fimmu.2022.931153 (PMC9344132; doi:10.3389/fimmu.2022.931153)
Supplement: Supplementary Table 1 — Respiratory status at referral for lung transplantation. Pulmonary function (spirometry, body plethysmography, diffusing capacity), exercise capacity test (6 minute walk distance (6MWD), cycloergometry) and arterial blood gas evaluation at referral for lung transplantation in 2016, demonstrating severe restrictive pulmonary dysfunction and decreased oxygen uptake, impaired exercise capacity and hypoxic, hypercapnic respiratory insufficiency. [file Presentation_1.pptx]

## Slide 1
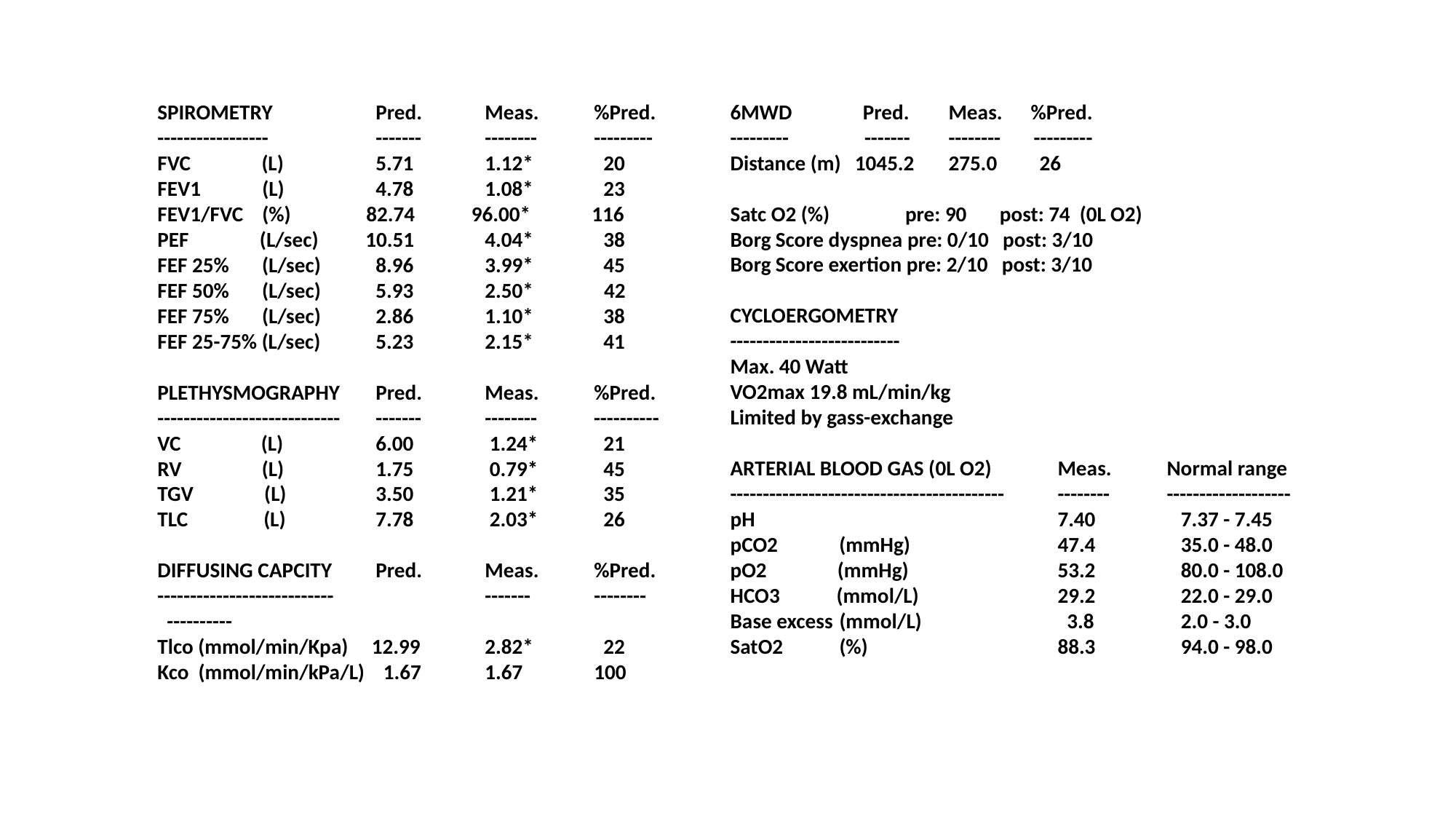

6MWD 	 Pred.	Meas. %Pred.
--------- ------- 	-------- ---------
Distance (m) 1045.2 	275.0 26
Satc O2 (%) pre: 90 post: 74 (0L O2)
Borg Score dyspnea pre: 0/10 post: 3/10
Borg Score exertion pre: 2/10 post: 3/10
CYCLOERGOMETRY
--------------------------
Max. 40 Watt
VO2max 19.8 mL/min/kg
Limited by gass-exchange
ARTERIAL BLOOD GAS (0L O2)	Meas.	Normal range
------------------------------------------	--------	-------------------
pH			7.40	 7.37 - 7.45
pCO2 (mmHg)		47.4	 35.0 - 48.0
pO2 (mmHg)		53.2	 80.0 - 108.0
HCO3 (mmol/L)		29.2	 22.0 - 29.0
Base excess	(mmol/L)		 3.8	 2.0 - 3.0
SatO2	(%)		88.3 	 94.0 - 98.0
SPIROMETRY 	Pred. 	Meas. 	%Pred.
----------------- 	------- 	-------- 	---------
FVC (L) 	5.71 	1.12* 	 20
FEV1 (L) 	4.78 	1.08* 	 23
FEV1/FVC (%) 82.74 96.00* 116
PEF (L/sec) 10.51 	4.04* 	 38
FEF 25% (L/sec) 	8.96 	3.99* 	 45
FEF 50% (L/sec) 	5.93 	2.50* 42
FEF 75% (L/sec) 	2.86 	1.10* 	 38
FEF 25-75% (L/sec) 	5.23 	2.15* 	 41
PLETHYSMOGRAPHY 	Pred. 	Meas. 	%Pred.
---------------------------- 	------- 	-------- ----------
VC (L) 	6.00 	 1.24* 	 21
RV (L) 	1.75 	 0.79* 	 45
TGV (L)	3.50 	 1.21* 	 35
TLC (L)	7.78 	 2.03* 	 26
DIFFUSING CAPCITY 	Pred. 	Meas. 	%Pred.
--------------------------- 	------- 	-------- ----------
Tlco (mmol/min/Kpa) 12.99 	2.82* 	 22
Kco (mmol/min/kPa/L) 1.67 	1.67 	100
